# The Molecular Genetics and Cellular Mechanisms Underlying Pulmonary Arterial Hypertension

**DOI:** 10.6064/2012/106576

**Published:** 2012-12-20

**Authors:** Rajiv D. Machado

**Affiliations:** School of Life Sciences, Faculty of Science, University of Lincoln, Brayford Pool, Lincoln LN6 7TS, UK

## Abstract

Pulmonary arterial hypertension (PAH) is an incurable disorder clinically characterised by a sustained elevation of mean arterial pressure in the absence of systemic involvement. As the adult circulation is a low pressure, low resistance system, PAH represents a reversal to a foetal state. The small pulmonary arteries of patients exhibit luminal occlusion resultant from the uncontrolled growth of endothelial and smooth muscle cells. This vascular remodelling is comprised of hallmark defects, most notably the plexiform lesion. PAH may be familial in nature but the majority of patients present with spontaneous disease or PAH associated with other complications. In this paper, the molecular genetic basis of the disorder is discussed in detail ranging from the original identification of the major genetic contributant to PAH and moving on to current next-generation technologies that have led to the rapid identification of additional genetic risk factors. The impact of identified mutations on the cell is examined, particularly, the determination of pathways disrupted in disease and critical to pulmonary vascular maintenance. Finally, the application of research in this area to the design and development of novel treatment options for patients is addressed along with the future directions PAH research is progressing towards.

## 1. Pulmonary Vascular Development

Three distinct models have been put forward in an attempt to shed light on the processes underlying the development of the pulmonary vascular bed and, in particular, the respective roles played by the key morphological events of vasculogenesis and angiogenesis. Vasculogenesis is defined by recruitment and differentiation of the endothelial progenitor cells into mature endothelial cells which proliferate, migrate, differentiate, and organise into a vascular plexus that forms the foundation for the early vascular system. Angiogenesis is the process of branching growth from existing vessels. Favouring angiogenesis as the predominant mechanism in pulmonary vascular development, Parera et al. suggest that expansion of the lung bud is resultant upon the formation of new capillary structures from previously established vessels [1]. By contrast, Hall et al. support vasculogenesis as the central process driving the generation of arteries and veins from the central vascular plexus [2]. Finally, deMello and Reid implicate both processes in vascular development, positing the theory that the early events involve the differentiation of mesenchymal cells to endothelial populations that is succeeded by angiogenic mechanisms impacting on endothelial cells derived from preexistent vessels [3].

The development of the pulmonary vascular system and circulation depends on an array of genetic factors working in a tightly regulated spatiotemporal fashion. These elements include transcription factors and cytokines amongst others. Among the key factors known to regulate vessel formation are the vascular endothelial growth factor (VEGF), transforming growth factor beta (TGF-*β*) family members, and angiopoietins along with several growth factor receptors [4]. Of these VEGF is intrinsic to pulmonary vascular development. Transgenic mouse studies wherein VEGF alleles have been conditionally ablated in the lung epithelium results in a phenotype exhibiting the near complete loss of the pulmonary capillary network [5]. In humans, heterozygous loss of VEGF leads to embryonic death and marked abnormalities of vascularisation [6]. VEGF is instrumental in generation of the vasoactive metabolites nitric oxide (NO) and prostacyclin (PGI_2_) via activation of endothelial nitric oxide synthase and prostacyclin synthase, respectively. Both metabolites are critical in the regulation of vascular tone [7, 8]. The TGF-*β* receptors and ligands are involved in lung morphogenesis and formation of the pulmonary circulatory system with the cytoplasmic mediators, the Smads, playing a role in pulmonary artery endothelial cell (PAEC) proliferation, and migration [9]. The angiopoietin family along with their receptor Tie-2 are important in endothelial cell migration and in the control of vascular maintenance and remodelling. Defects in these proteins results in extreme vascular abnormalities [10].

### 1.1. Haemodynamic and Structural Adaptation in Foetal and Neonatal Pulmonary Circulation

**Figure 1:**
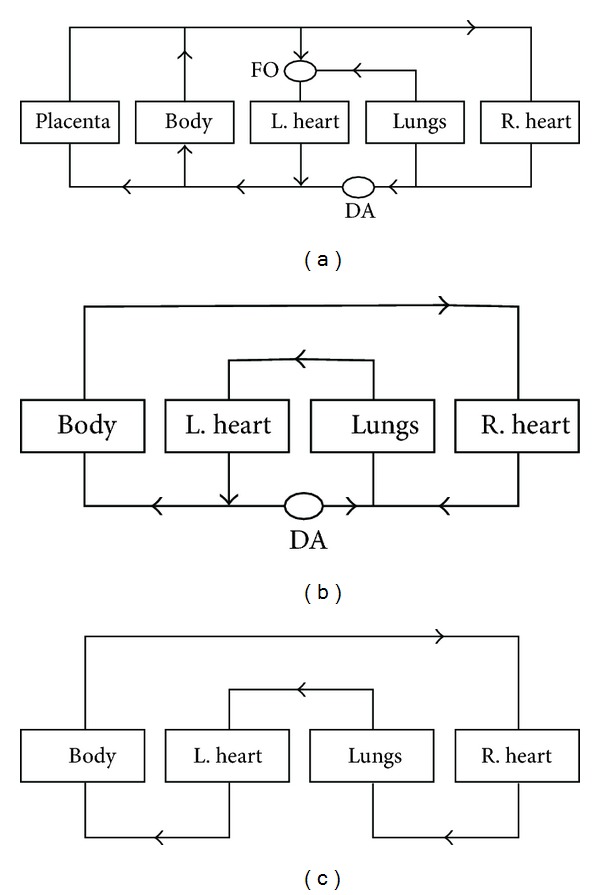
Development of the circulatory system. Systemic and pulmonary circulation in the (a) foetus, (b) neonate, and (c) adult. The arrows refer to the direction of blood flow through the anatomical compartments, depicted by the labelled rectangular boxes. The oval shapes labelled FO and DA refer to the foramen ovale and ductus arteriosus, respectively.

The site of blood oxygenation in the foetus is the placenta. At this stage of life the patent foramen ovale (FO) and the patent ductus arteriosis (DA) are open. The flow of blood from the placenta is, thus, directed away from the foetal lungs. Oxygenated blood is directed to the chambers of the heart via the foramen ovale. Negligible quantities of blood enter the lungs as the high resistance of foetal pulmonary vasculature encourages diversion through the ductus arteriosis. The resistance relates to the morphology of the muscular arteries and arterioles, in which the smooth muscle layer is thick and the lumen narrow [11–14]. Upon birth, the lungs are employed in blood oxygenation. A fall in pulmonary vascular resistance soon follows as a consequence of pulmonary artery and arteriole dilatation concomitant with an increase in systemic pressure. Upon systemic pressure surpassing that of pulmonary vascular resistance the foramen ovale shuts, soon followed by the contraction of the ductus arteriosis [15, 16] (Figures 1(a) and 1(b)).

### 1.2. Haemodynamic and Structural Features of Postnatal and Adult Circulation

The closure of the ductus arteriosis and, subsequently, the foramen ovale generates a dramatic increase of blood flow through the lungs (Figure 1(c)). During the first two or three weeks of life, significant structural changes to the muscular arteries and arterioles occur. Considerable luminal widening with accompanying medial thinning is exhibited. A more gradated decrease in media width progresses over one and a half years until the pulmonary vasculature reaches a steady adult state. Critical to the significant decrease in the postnatal circulation and, indeed, the maintenance of the adult circulation is the change in functional outcomes of signalling pathways comprising key vasoactive elements. The adult pulmonary circulation reflects its structural features by being a low-pressure system of between 7 and 15 mm Hg with an average of approximately 9 mm Hg [17–20].

## 2. Pulmonary Arterial Hypertension

Pulmonary arterial hypertension (PAH) represents a dramatic reversal of the physiological adult circulation to a disease state bearing features of foetal high vascular resistance. Whilst the disorder is uncommon with an incidence calculated at 1-2 cases per million per year, it is progressive and typified by high morbidity and mortality. PAH is clinically diagnosed when mean pulmonary artery pressure is maintained at >25 mm Hg at rest or 30 mm Hg during exercise with normal pulmonary capillary wedge pressure and elevated pulmonary vascular resistance [21]. Diagnostic protocols include patients having a Doppler echocardiogram to determine right heart size and function, left ventricular diastolic and systolic function, and putative causes of pulmonary hypertension [22]. Prior to a treatment course being decided patients require right heart catheterization to measure hemodynamic parameters, for example, pulmonary arterial pressure and vascular resistance [23]. PAH afflicts both adults and children but, in the main, occurs in the 3rd to 4th decade of life. For reasons that are yet to be fully explained the disease exhibits a sex bias affecting twice as many females than males [24]. A proportion of PAH patients, approximately 10%, have a family history of disease which in certain cases has been observed to be transmitted through multiple generations [25]. Due to the advent of contemporary therapies, patient life expectancy has been improved but the disease remains incurable. In the absence of heart-lung transplantation the disease, typically, remains fatal with death occurring due to the strain imposed on the right side of the heart. A significantly lower number of male offspring of PAH affected women has been noted, suggestive of selective male foetal wastage. The condition appears to affect all racial and ethnic groups equivalently [24].

### 2.1. Classification of Pulmonary Hypertension (PH)

As defined by the 2008 World Health Organization symposium on pulmonary hypertension, there exist 5 major classes. These comprise: Group 1. PAH; Group 2. PH associated with left heart disease; Group 3. PH associated with alveolar hypoxia and/or other disorders of the respiratory system; Group 4. Thromboembolic disease resulting in PH; Group 5. PH with unclear multifactorial mechanisms. PAH itself is sub-divided into 5 additional categories comprising idiopathic PAH (IPAH), heritable PAH (HPAH), which identifies patients with multiple affected family members and/or an identified germ-line mutation, PAH associated with known risk factors for disease, PAH associated with additional diseases (APAH) which may include HIV infection, systemic sclerosis, and congenital heart disease. The final class is persistent pulmonary hypertension of the newborn [26].

### 2.2. Functional Classes of Pulmonary Arterial Hypertension

The World Health Organization, in addition to the clinical classification of PH, has developed a four tier classification system that segregates patients on the basis of disease severity with Class I being the mildest and Class IV the most severe form of disease. Patients falling within Class I comprise individuals with PH that is not accompanied by constraints on physical activity. Moreover, normal physical activity does not result in dyspnoea, fatigue, syncope, or chest pain. Class II patients have PH with mild limitation of physical activity whereas Class III patients are severely impaired in the ability to perform physically exerting tasks. Class IV patients present with symptoms if any physical activity is performed; these individuals are representative of end stage disease with clear indications of heart failure. Additionally, shortness of breath and general fatigue are evident in a resting state [27].

### 2.3. Histopathology of PAH

**Figure 2:**
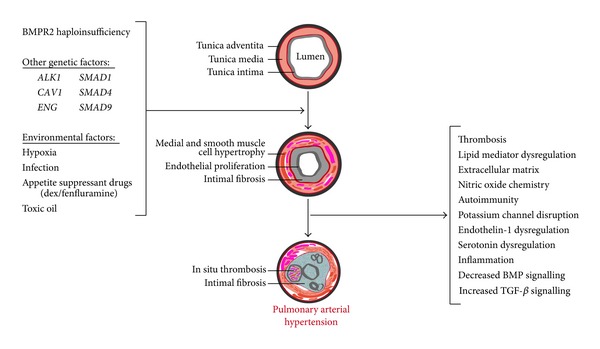
Cause and effect in pulmonary arterial hypertension. Schematic representing the genetic and environmental factors involved in the aetiology of PAH and the biochemical readouts associated with PAH presentation. *BMPR2* haploinsufficiency remains the major genetic contributant to disease pathogenesis. The central depiction of a section through a pulmonary artery shows the hallmark features at each stage of PAH progression.

PAH is associated with the development of vascular lesions and aberrant vascular re-modelling. While a series of lesions are typically observed in PAH, these can be present as heterogeneous populations both within and between families suggesting that the initial causal factor generating lesion types is the same. The arterial circulation is predominantly impacted upon although there is evidence of limited venous remodelling. A frequent pathological observation in PAH is medial hypertrophy of the muscular pulmonary arteries and muscularization of the arterioles. Accompanying this is a duplication of the elastic lamina and a characteristic thickening of the medial smooth muscle (Figure 2). Affected vessels are similar to foetal muscular arteries albeit in an adult circulation system. A hallmark pathological finding in PAH is the plexiform lesion present in approximately a third of patient lung tissue. Plexiform lesions are typically associated with end-stage PAH but are not emblematic of the disease as they are also observed with other disorders arising with PH. Situated in muscular arteries, usually of an external diameter between 100–200 *μ*m, they typically arise at the origin of pulmonary arterioles and display aberrations of the media and intimal lining. The lesion is defined by the segmental destruction of the medial cell wall layer. The lesion typically presents with a profusion of smooth muscle cells, myofibroblasts, macrophages, and proliferating endothelial cells. The upregulation of growth factors, such as vascular endothelial growth factor (VEGF) and its receptor, has been described in the endothelial cells within the plexiform lesion [28–30]. The initiating factors and, indeed, cell type responsible for the generation of plexiform lesions remain a source of uncertainty. While debate continues as to the source of lesional formation being smooth muscle cells or endothelial cells an important observation has been the report of endothelial cells within lesions being monoclonal, suggestive of neoplastic expansion. Furthermore, plexiform endothelial cells from idiopathic patients have been shown to exhibit microsatellite instability in DNA repair genes, pro-apoptotic genes and in the *TGF-*β** gene, which is also a central feature of cancer cells and is described as the “two hit” tumour suppressor gene hypothesis [31]. Contrastingly, however, cells obtained by microdissection of plexiform lesions from lungs of HPAH subjects exhibited no evidence of similar somatic mutations. These data indicate that microsatellite instability is unlikely to underpin both categories of PAH [32]. The arterial lumen, proximal to the lesion, is obstructed by eccentric or concentric intimal fibroelastosis. Distally, the artery is markedly dilatated and thin-walled.

### 2.4. Disease Pathogenesis

#### 2.4.1. Endothelial Cell Abnormalities

Imbalances in vasoactive metabolites are a central feature of the vascular endothelium of PAH patients. Vasocontrictive agents, for example, thromboxane and endothelin-1, are found to be significantly upregulated in pulmonary arteries [33, 34]. By contrast, the vasodilators prostacyclin, nitric oxide, and the nitric oxide messenger cyclic guanosine monophosphate are present at much reduced levels. Through unclear mechanisms, the upstream catalysts of these proteins, prostacyclin synthase, and nitric oxide synthase, respectively, are also greatly diminished in patient lung tissue [35, 36].

#### 2.4.2. Inflammation

Inflammation has long been recognised as having a role in the development of PAH. Evidence of heightened levels of inflammatory cytokines in patient tissue include tumour necrosis factor, IL-6, IL-1*β*, and monocyte chemoattractant protein 1 [37, 38]. Vascular lesions in PAH patient lung display classic inflammatory features which include the presence of macrophages, monocytes, and T and B lymphocytes [39]. Inflammatory chemokines are also found at elevated levels in PAH patients, in particular the chemotactic protein FKN, RANTES, and chemokine ligand 5 [40].

#### 2.4.3. Voltage-Gated Potassium Channels

Intracellular calcium levels are regulated by voltage-gated potassium channels which act by regulating membrane potential and controlling the release of calcium. Intracellular calcium is integral in driving contraction of pulmonary artery smooth muscle cells (PASMCs) and promoting hypertrophy. Calcium levels in PAH smooth muscle cells have been shown to be elevated likely as a direct consequence of the observed reduction in transcript for potassium channels and low current in patients by comparison to controls. The paucity of potassium channels has been posited as a potential explanation for the vasoconstriction and uncontrolled proliferation observed in patients [41].

#### 2.4.4. Serotonin and the Serotonin Transporter

IPAH patients exhibit elevated levels of serotonin (5-HT) which persist post heart-lung transplantation. This finding indicates a causal role for 5-HT in the pathogenesis of PAH [42]. 5-HT, stored primarily in the platelets, is a potent vasoconstricting agent and mitogen which promotes smooth muscle cell proliferation. The mitogenic effects of 5-HT are mediated by the serotonin transporter, 5-HTT [43]. Indeed, chemical inhibition of 5-HTT results in a loss of 5-HT induced cell proliferation. 5-HTT is expressed at high levels on PASMCs and increased production of 5-HTT in PASMCs is capable of triggering spontaneous PAH [44].

#### 2.4.5. The Warburg Phenotype

Cancer cells display dysmorphic mitochondria and a glycolytic shift in metabolism which is believed to confer resistance to apoptosis [45]. This cellular phenomenon, known as the Warburg phenotype, has also been observed in PASMCs and PAECs from human PAH patients as well as in a rodent model of PAH [46]. This link to a cancer phenotype appears to be associated with depleted levels of the tumour suppressor gene and antioxidant enzyme SOD2. SOD2 scavenges super-oxide, converting it to hydrogen peroxide in the mitochondria, a critical step in the regulation of cell proliferation. Significant downregulation of SOD2 is observed in the pulmonary arteries and plexiform lesions of PAH patients [47].

#### 2.4.6. Extracellular Matrix

Mitogens in the extracellular matrix (ECM) are released and tenascin-C upregulated due to the abnormal degradation of the ECM by matrix metalloproteinases and elastases in patients. Tenascin-C has a proproliferative effect on smooth muscle cells as it enhances the response to mitogenic agents. Studies have shown that inhibition of these substances reduced vascular remodelling and the severity of disease thereby indicating their importance in smooth muscle homestasis [48] (Figure 2).

## 3. Genetics and Genomics of PAH

Heritable PAH was first reported by Dresdale et al. in 1951 [49]. In families, the disease segregates in an autosomal dominant manner and with markedly reduced penetrance as indicated by the absence of overt disease symptoms in multiple obligate carriers. Although all examined pedigrees suggest that HPAH is a single gene disorder, the reduced penetrance, sex bias, and disparity of age of disease onset both within and between families serve to highlight complex traits of inheritance [50]. Using microsatellite based genotyping and standard linkage analysis in multiple families, a locus for the causative gene was identified by two independent groups in 1997 [51, 52]. Nichols et al. employed 6 large pedigrees for linkage studies and isolated a minimal linkage interval of 25 cM with a maximum LOD score of 6.97 [52]. This large region was saturated with further markers of high informativity and haplotype mapping was conducted in several additional kindreds to refine the linkage peak by the fine mapping of recombination events amongst affected individuals. These genetic analyses facilitated the refinement of the original 25 cM interval to a more tractable 4.8 cM located on chromosome 2q33 [53]. Although typically a late-onset autosomal dominant disease, microsatellite data generated during the course of the linkage investigation did not provide evidence of shared disease haplotypes in US and UK populations that would be indicative of the presence of founder mutations.

### 3.1. Transcript Mapping and Positional Cloning of the Gene Underlying HPAH

To derive a clear appreciation of the actual size of the interval and unambiguously position polymorphic markers and, critically, expressed sequence elements a physical map was created employing contigs comprising YACs, BACs, and PACs. These studies established the interval as being 5.8 Mb and harbouring within it 79 transcriptional units [53]. Characterized genes with likely biological relevance to PAH were prioritized for analysis by direct Sanger sequencing. Screening of a number of candidate genes culminated in the identification of independent heterozygous mutations, in numerous familial cases, of the gene *BMPR2* [54, 55]. *BMPR2* encodes the transmembrane bone morphogenetic receptor type II of the TGF-*β* superfamily of signalling molecules. TGF-*β* molecules are indispensible in a multitude of cellular activities which include proliferation, apoptosis, migration and differentiation [56]. Shortly after the identification of *BMPR2* defects in PAH families, mutation analysis was extended to a cohort of patients with sporadic onset of disease. All cases were rigorously screened to ensure the absence of a family history, associated disease and exposure to known risk factors. Initial investigations revealed the presence of *BMPR2* mutations in 26% of patients thereby expanding a role for this gene in the wider spectra of PH. Thereafter, several international studies have been conducted on IPAH patients with mutation detection rates ranging from 11–40% [57–60].

### 3.2. *BMPR2* Structure and Function

*BMPR2* is a relatively large gene, consisting of 13 coding exons arrayed over 190 kb of genomic DNA with a transcriptional start site at base pair position −1148 relative to the initiation codon adenine. It has an unusually long 3′ UTR of approximately 11 kb [61]. The transcript encodes a protein of 1038 amino acids comprising an extracellular ligand binding domain (exons 2 and 3), a single pass transmembrane domain (exons 4 and 5), a highly conserved catalytic kinase domain (exons 6 to 11) and a cytoplasmic domain (exons 12 and 13) of uncertain functional relevance. Of note, this cytoplasmic tail is unique in length amongst other TGF-*β* receptors all of which contain short intracellular tracts. Exon 12 is removed by alternative splicing to render a naturally occurring shorter receptor species which while, as is the case with the long form, is ubiquitously expressed at the transcript level, the protein product has not been extensively tested to determine functionality [62].

The BMP signalling pathway is a highly regulated system controlled on multiple layers and in a cell-specific manner by a variety of mechanisms that include competitive ligand binding, intracellular inhibition of receptor types and cell-specific utilisation of different receptor combinations to generate myriad signalling outcomes. In its basic form, signalling is initiated at the cell surface when extracellular ligand is bound by a heterotetrameric complex comprising BMPR-II and a type-I partner receptor which may be BMPR1A or BMPR1B [63]. As an illustration of pathway specificity, in pulmonary endothelial cells, BMPR-II binds the type I receptor ALK-1 which is predominantly expressed in the endothelium in order to transduce signal in response to the ligand BMP9 [64]. When brought together upon binding ligand the constitutively kinase active BMPR-II transphosphorylates the GS domain of its type I partner thus activating its catalytic domain. The type I receptor, next, contacts and phosphorylates cytoplasmic effector molecules, for example, the receptor Smad proteins (R-Smads). BMP signalling is differentiated from TGF-*β* pathways by the fact that each activate a different subset of R-Smads. In the case of the BMPs, these are R-Smad1, 5, and 8 whereas the TGF-*β* receptors phosphorylate Smads 2 and 3. Common to both systems is Smad4 which binds the activated R-Smads and chaperones them to the nucleus where, in combination with nuclear cofactors they act, in complex, to regulate the transcription of a target set of genes [63]. More recently, it has become evident that signalling routed through this pathway is far more complex than previously thought. Several Smad independent pathways have emerged from the characterization of BMPR-II interacting proteins, for example, Tctex-1, LIMK-1, RACK-1, tenascin-1, that regulate cellular processes which include actin cytoskeletal dynamics and maintenance of the extracellular matrix [65–68]. An important pathway regulated by BMP signalling is that of p38MAPK [69]. Increasingly, an understanding has grown of the mechanisms governing p38MAPK activation downstream of BMPR-II and, indeed, Smad activation itself. Optimal signalling through either downstream pathway appears to be dependent on endocytosis of receptor complexes. Smad signalling requires receptor internalization through clathrin-coated pits whereas p38MAPK activation is reliant on receptor complexes being endocytosed through a caveolae mediated route [70]. The mode of receptor oligomerization is also considered vital in determination of downstream pathway activation. Receptor complexes may either be preassembled or induced by ligand; in the former instance Smad signalling is the favoured pathway whereas ligand recruitment of receptor complexes, the more common mechanism, leads to alkaline phosphatase activation via the p38MAPK pathway [71].

### 3.3. The *BMPR2* Mutation Spectrum in HPAH

Mutations in *BMPR2* have been now reported in a multitude of ethnic groups as a result of a systematic survey of exons, intron-exon junctions, and, less frequently, noncoding regions including the 5′ and 3′ UTR and putative promoter region. In the main, point mutations in the gene have been examined using Sanger sequencing and/or denaturing high-performance liquid chromatography (DHPLC). Large scale gene rearrangements have been screened for using multiplex ligation-dependent probe amplification (MLPA) technology in addition to conventional Southern blotting. These independent investigations, when combined, identify deleterious variation in 70% of subjects with affected relatives, with more likelihood to be found when gene screening routinely advances to analysis of intronic and regulatory regions. More unexpectedly, mutations of genes encoding BMPR-II and, indeed, other members of the TGF-*β* superfamily have been detected in PAH associated with other conditions or risk factors, for example exposure to the appetite suppressant drugs fenfluramine and dex-fenfluramine. The distribution and class of mutation in hereditary and spontaneous disease is largely indistinguishable. In both HPAH and IPAH, mutations are arrayed across the gene, albeit at extremely low frequency in exons 5 and 13, and comprise all categories including missense, nonsense, frame-shift, splice-site, small deletions and insertions, as well as larger gene rearrangements. To date, over 300 independent mutations of *BMPR2* have been identified [72]. In the main, the variant level of severity has been inferred by confirmation of segregation amongst affecteds and carriers in families, degree of evolutionary conservation across species and occurrence in key functional domains. However, representative examples of mutant alleles, impacting upon the major receptor domains, have been taken forward by several research groups to *in vitro* functional analysis of the effect of identified patient specific mutations on downstream signalling pathways.

#### 3.3.1. Haploinsufficiency Is the Primary Molecular Mechanism of Disease

Mutations of *BMPR2* likely to cause premature truncation of the open reading frame constitute large gene deletions and duplications (~6%), splice-site mutation (~9%), frame-shift (~24%), and nonsense defects (~29%). Thus, 70% of all identified mutations in HPAH would be predicted to lead to aberrantly truncated mutant alleles [72, 73]. Truncating mutations have been detected across the gene but spare the terminal exon 13. The effect of truncating point mutations on messenger RNA was assessed using patient cell lines harbouring this class of mutation. The level of the transcript under physiological conditions was estimated by means of quantitative polymerase chain reaction (qPCR) assays. As predicted, upon the addition of puromycin, a well known inhibitor of the nonsense mediated decay (NMD) pathway, transcript levels increased significantly. These data indicate that the majority of mutations predicting premature truncation are detected by the NMD surveillance machinery and removed from the cell by degradation of the mutant harbouring allele [74]. Taken together with the evidence of large and whole gene deletions of *BMPR2*, these observations firmly establish haploinsufficiency as the central molecular mechanism of disease in familial and sporadic forms of PAH [75] (Figure 2). While the vast majority of truncating mutations typically impact upon the coding region an interesting exception is found in the form of a double substitution of contiguous bases approximately 900 base pairs upstream of the start site of translation. The GC < AT mutation was identified in a large kindred and observed to segregate with the disease phenotype. *In silico* analysis of the region of sequence harbouring this variation suggested the introduction of a new translational start site stronger than the wild-type which, if employed, would lead to the incorporation of a premature termination codon in exon 1 of the gene. As this event would be predicted to produce a transcript susceptible to NMD, allele-specific polymerase chain reaction was employed to test transcript levels from patient cell lines before and after the application of NMD inhibitors. The resultant data indicated a consequent restoration of mutant message indicating that NMD was being triggered. The detection of this novel mutation serves to emphasise the likely presence of pathogenic variation contained within noncoding sequence not typically screened [61].

#### 3.3.2. Amino Acid Substitution in HPAH

Missense mutations underlying amino acid substitutions impact primarily on areas of the receptor that are of pivotal catalytic and/or structural importance. As such, this form of mutation has been found to be largely restricted to those exons and, indeed, residues that are pre-requisites for BMPR-II signalling activity. The majority of missense mutations have been identified in exons 2 and 3 encoding the extracellular domain, and exons 6 to 11, encoding the kinase domain. Only a handful of missense mutations have been identified in exon 12, responsible for the long cytoplasmic tail [72, 73].

#### 3.3.3. Mutations of the BMPR-II Extracellular Domain

Three dimensional structural studies reveal that the extracellular domains of the TGF-*β* receptors, including BMPR-II, are highly organised structures which bear resemblance to a class of toxins known as the three finger toxin family. This complex fold is substantially dependent on the formation of five disulphide bridges themselves dependent on 10 cysteine residues spread across the proximal exons. The importance of these particular residues is indicated by the fact that they are invariant in the majority of type II receptor species [76]. Of note, amino acid substitutions within this domain predominantly affect the conserved cysteine residues, testimony to their importance to receptor integrity. *In vitro* studies using tagged constructs bearing cysteine mutations demonstrated that the subcellular localisation of the encoded mutant proteins was cytosolic by contrast to the plasma membrane positioning of the wild-type receptor. These results point to the likelihood of normal membrane trafficking being compromised as a consequence of protein misfolding leading to the trapping of mutant receptors in the cytoplasm [77].

#### 3.3.4. Kinase Domain Defects in PAH

The kinase domain of BMPR-II belongs, along with the other TGF-*β* receptors, to the extensive and highly evolutionarily conserved eukaryotic protein kinase (EUK) superfamily. Eukaryotic kinases share structural and functional features as well as the phosphate group transfer mechanism. EUKs are composed of 12 subdomains each with specific functional characteristics necessary to effect the step-wise processes required for a successful catalytic outcome. Broadly, the N-terminal end of the receptor is responsible for adenosine triphosphate binding while subdomains in the distal half are required for substrate recognition of initiation of substrate phosphorylation. Within these subdomains are distinctive patterns of conserved residues and, further, as revealed by X-ray crystallography, invariant residues which are indispensible to function [78]. Missense mutations typically cluster within the more highly conserved subdomains and conserved residues. This is best illustrated by recurrent PAH-specific mutation at position 491 in the amino acid chain. Within the kinase domain this residue is critical to activity as it forms an ion pair with a conserved glutamic acid at position 386. By contrast, few variants, the majority of which may be described as being of unknown significance, arise in the weakly conserved regions of the kinase domain, for example, subdomain IV, which does not appear to play a major role in kinase function or structure [72, 73]. All noncysteine substitutions of the kinase domain are localised correctly to the plasma membrane due to the structural integrity of the receptor being preserved. Transient transfection of a large series of mutant receptors harbouring kinase domain defects indicate the loss of kinase function by their inability to activate a Smad binding element upstream of a luciferase reporter gene. In many cases the receptor was effectively kinase dead by comparison to wild-type controls. Intriguingly, it was also noted that transfection of both cysteine and noncysteine receptors lowered luciferase readouts to below base-line levels implying a dominant negative effect on the wild-type receptor. This was hypothesised as being a result of sequestration of the wild-type partner by mutant receptors into signalling defective complexes. However, it is often the case that assays of this nature do not accurately reflect the *in vivo* situation making over interpretation of the data unwise [77, 79].

#### 3.3.5. Mutations of the Cytoplasmic Terminus

Approximately 15% of the mutation load in PAH is confined to the cytoplasmic tail region with the vast majority of recorded mutations in this domain truncating in nature [72, 73]. Of interest, the small number of missense mutations observed in patients are clearly distinct from the rest of the spectrum. These mutant receptors appear broadly analogous to wild-type in the ability to translocate to the cell-surface and activate luciferase reporters [77, 79]. To investigate the pathogenicity of these variants, research streams turned to identifying Smad-independent pathways, regulated by the cytoplasmic domain, that were potentially of significance in disease precipitation and progression. To facilitate this objective, a number of studies focussed on developing an understanding of the role of the cytoplasmic tail in signalling by the identification of protein interacting partners. Amongst the interacting proteins identified were the cytoskeletal proteins Tctex-1, a light chain of the macromolecular motor cytoplasmic dynein and LIMK-1, a regulator of actin polymerization. Tctex-1 was discovered to be a kinase substrate of BMPR-II; the presence of mutation in the cytoplasmic tail attenuated this process. Mutations in this domain resulted in the constitutive activation of LIMK-1 resulting in abnormalities in actin dynamics [65, 66]. More recently, studies conducted on a transgenic mouse harbouring a cytoplasmic domain mutation (p.R899X), which due to massive overexpression of the transgene acts in a dominant negative manner, demonstrated augmented levels of Rho/Rho-kinase. Moreover, in certain cell populations this resulted in increased production of IL-6 which, when suppressed, ameliorated symptoms of disease. No perturbation of the Smad signalling pathway was observed suggesting that activation of Rho-kinase may represent an additional disease pathway specifically related to the cytoplasmic tail [80].

#### 3.3.6. Pathway Dysregulation as a Result of BMPR-II Mutation

The single pathway disrupted by all mutation irrespective of position is that of the proproliferative kinase p38MAPK which is found to be upregulated by BMPR-II mutation. p38MAPK has been linked, *in vitro*, to the abnormal proliferation of PAH cells as this cellular defect can be reversed in the presence of specific p38MAPK inhibitors [77].

TGF-*β* signalling in normal PASMCs exerts an antiproliferative response. By contrast, BMPR-II defects in human and experimental PAH appear to induce an upregulation in TGF-*β* signalling in concert with a proproliferative effect on PASMCs. As examination of the canonical TGF-*β* pathway did not uncover evidence of dysregulation, it was speculated that Smad-independent signalling might play a role. Assessment of mutant cell lines with identified *BMPR2* mutation led to the discovery of an elevation in the expression and activity of TGF-*β* activated kinase (TAK-1). As the downstream substrates of TAK-1 include p38MAPK and ERK-1, it has been posited that the increased pool of TAK-1, responding to TGF-*β* stimulation, drives the uncontrolled proliferation of mutant cells by activation of its substrates [81]. Davies et al. drew a link between BMPR-II dysfunction and misdirected NF-*κ*B signalling leading, in turn, to an increased production of IL-6 and PASMC proliferation in response to abnormally elevated TGF-*β* stimulation [82].

#### 3.3.7. Atypical *BMPR2* Mutations in APAH

The majority of mutations observed in studies conducted on PAH associated with other conditions and/or risk factors, primarily appetite suppressant drugs, demonstrate a marked dissimilarity to the HPAH mutation spectrum. In contrast to HPAH, over 90% of these variants are missense mutations which display a different profile to the amino acid substitutions in hereditary and spontaneous disease. Whereas cysteine residues are predominantly mutated in HPAH, these residues are spared in PAH associated with congenital heart disease and anorexigen intake. Moreover, many of the identified substitutions occur in regions of the receptor that make a limited contribution to the functional and structural integrity of the receptor, for example exon 5 of the gene. *In vitro* reporter based studies aimed at determining the effect of a series of these reported APAH mutations on BMPR-II signalling activity concluded that their ability to activate the reporter gene was comparable to wild-type. Hence, interpretation of the mechanisms by which these variants trigger PAH is problematic. It is feasible that these alleles represent low penetrance risk factors that require additional environmental insults and genetic modifiers to precipitate disease [72, 73].

#### 3.3.8. Recurrent *BMPR2* Mutations in HPAH

Mutations observed on multiple occasions in unrelated cases accounts for approximately 40% of observed variation in HPAH and are comprised of at least 25 independent defects. While large-scale systematic studies have not been performed to determine whether these mutations arise from common founder haplotypes, those mutations which have been analysed (c.994C>T (p.R332X), c.2579delT (p.N861fsX10) and c.2695C>T (p.R899X)) by microsatellite marker haplotyping clearly confirm that in each case the mutation was harboured in a distinct haplotype with no evidence of marker sharing between patients [72, 73]. These results support the hypothesis of a recurrent mutation process in favour of the presence of ancestral mutations ascertained in multiple individuals. Moreover, the majority of the observed recurrent mutations occur at CpG islands and, thus, likely arise as a result of spontaneous deamination of methylated cytosines with subsequent conversion to thymidine, a relatively common mutational mechanism. Recurrent frameshift mutations as a result of small insertions and deletions are typically found in regions of low sequence complexity, potentially implicating replication slippage as a cause for mutation at those positions. Investigation of isolated populations, notably that of Finland, also failed to uncover evidence of founder mutations indicating that if it does exist it is most likely uncommon [83].

#### 3.3.9. Genotype-Phenotype Correlations

Analyses of a relationship between mutation class and position with broad disease parameters including age of onset, hemodynamic function in patients, and survival failed to yield data indicative of a meaningful correlation. More recently, research into putative correlations has endeavoured to interrogate more subtle aspects of phenotype. Elliott et al. attempted to assess the relationship between *BMPR2* sequence variation and vasoreactivity, an important clinical consideration in the treatment of patients using vasodilators such as prostacyclin analogues. A total of 67 familial and idiopathic patients were investigated for *BMPR2* nonsynonymous variation, of which 27 patients proved positive for variation with 22 harbouring bona fide pathogenic mutations. Vasoreactivity was evaluated between the sets of patients with and without variation using stringent clinical criteria. None of the mutation carriers were found to be vasoreactive by contrast to 35% of patients with no form of variation. These results are a compelling indication of a correlation between defective BMPR-II signalling and vascular tone and responsiveness [84].

In a Chinese study comprising 305 individuals, the presence of *BMPR2* mutations was noted to be associated with higher risk of mortality once adjusted for age and gender. The effect was most prominent in the male group but did not reach statistical significance in females which the authors attributed to the more complex disease aetiology in women [85].

In seeking to explore the reasons behind the remarkably low penetrance in *BMPR2* mutation carriers Cogan et al. examined the role played by alternatively spliced isoforms of the gene. A comparative analysis was conducted between lymphocytes from 47 affected mutation carriers versus 35 unaffected mutation carriers. Patients were determined to have a significantly higher ratio of the allele lacking exon 12 to the full-length transcript by comparison to carriers. Scrutiny of exon 12 revealed the presence of an exonic splice enhancer capable of binding serine/arginine-rich splicing factor 2 (SRSF2) which promotes exon inclusion. SRSF2 levels were found to be lower in patients than carriers and, theoretically would be anticipated to result in an elevation of levels of the shorter isoform [86].

### 3.4. Locus Heterogeneity in PAH

#### 3.4.1. ALK and Endoglin

PAH, infrequently, may copresent with the vascular disorder hereditary haemorrhagic telangiectasia (HHT) wherein patients have PAH clinically and histopathologically identical to the hereditary form of disease, although PAH can also develop as a direct consequence of the pathophysiology of HHT. HHT, or Rendu-Osler-Weber syndrome, is an autosomal dominant disease with an incidence of approximately 1/10,000 in Europe. HHT is characterised by abnormal vascular development and a range of distinctive clinical complications. An early, common symptom is epistaxes (nosebleeds), observed in up to 90% of affected individuals. Other clinical manifestations may include telangiectases, red to violet lesions that develop on digits; the facial, nasal, and buccal mucosa; and the gastrointestinal (GI) tract. Haemorrhage from the GI bed, noted in 20–40% of patients, is progressive and can result in chronic anaemia [87].

Arteriovenous malformations (AVMs) engineer a direct communication between the arterial and venous systems that bypasses the capillary bed. AVMs have been detected in the pulmonary circulation in 25% of HHT cases; the hepatic circulation in 8–16% of patients and the cerebral circulation in 15% of the affected population. AVMs are considered to be a major contributant to morbidity and mortality in HHT patients [87].

Although the primary pathophysiological defect in HHT has yet to be determined, genetic linkage studies have revealed molecular heterogeneity in HHT and the identity of two loci. The disease genes at chromosome 9q34 and 12q13 have been identified as encoding the TGF-*β* pathway receptors *endoglin* and *ALK1,* respectively [88, 89]. Non-linkage to chromosome 9 and 12, in two HHT pedigrees, has suggested the likelihood of a third, as yet unidentified, locus [90]. *Endoglin* is a 14 exon gene, expressed at high levels on human vascular endothelial cells. Disparate mutations have been recorded in *endoglin* and appear to correlate to a severe clinical phenotype, with a high incidence of pulmonary AVMs (PAVMs) [91].

*ALK1 *is organised into 10 exons with the first translated methionine within exon 2. Families, with segregating *ALK1* mutations, tend to exhibit late-onset and milder disease manifestation. PAVMs have never been reported in patients bearing mutations in this gene. The molecular basis of dominance, in both *endoglin* and *ALK1*, is widely accepted as being that of haploinsufficiency [92].

ALK-1 and endoglin are type I and type III receptors respectively operating within the TGF-*β* pathway. This biological link with BMPR-II suggested a common molecular aetiology in patients with PAH-HHT. Five pedigrees in which both diseases were cosegregating were analysed for mutations in the three genes encoding these receptors. Screening of the coding regions and intron/exon boundaries revealed the presence of two truncating mutations and three missense mutations in *ALK1*, thereby identifying a second gene predisposing to the development of pulmonary hypertension [93]. Since the original report of *ALK1* as an underlying factor in PAH, several groups have analysed additional cohorts and thereby built on the mutation spectrum of the gene. These studies now reveal that 20% of all known *ALK1* mutations lead to the onset of PAH and, of these, 81% are distinct to defects causing HHT alone. Of the identified mutations, amino acid substitutions constitute the predominant class and affect functionally key domains of the receptor, in particular the kinase domain and NANDOR box [94]. More recently, evidence has emerged of *ALK1* mutations causing PAH in the absence of HHT. However, it must be noted that these subjects, in the main, have early-onset or childhood PAH thus retaining the possibility that HHT could develop at a later stage [95]. *Endoglin* mutation may also represent a rare cause of PAH-HHT with four defects detected in patients thus far. All *endoglin* mutations would be expected to lead to premature truncation of the transcript [73].

#### 3.4.2. *SMADs* 1, 4, and 9

The discovery of mutations in three members of the TGF-*β* family leading to susceptibility to PAH emphasizes the role played by this signalling pathway in the maintenance of the pulmonary architecture. A number of recent studies have sought to expand on these findings by examining members of the downstream pathway for deleterious variation in patients in whom the known risk factors have been eliminated. By means of a direct sequencing approach in a limited cohort of 23 Japanese cases, Shintani et al. isolated a mutation in the BMP-specific receptor Smad gene *SMAD9* (p.C202X) predicting the incorporation of a premature truncation codon [96]. In the largest candidate gene study conducted in PAH a total of 324 idiopathic and associated PAH patients, previously excluded for mutations in *BMPR2*, *ALK1,* and *endoglin*, were screened for *SMAD1, -4, -5,* and -*9* by Sanger sequencing. This analysis resulted in the detection of 4 variants in the IPAH panel consisting of 198 cases, each of which was absent in a panel of 1000 controls and 60 fully sequenced exomes. These variants in *SMAD1* (p.V3A), *SMAD4* (p.N13S; c.1448-6T>C), and *SMAD9* (p.K43E) may define an additional 2.2% of the IPAH population with a genetic background to disease. However, whilst significant, it was found that the impact of these variants on functional outcomes was less extreme than classical *BMPR2* mutation. Therefore, they fall into the category of variants of unknown significance that, potentially, require other environmental and genetic factors to prove pathogenic. Alternatively, these defects may impact upon Smad-independent pathways, the investigation of which was not within the scope of this study [97]. Of interest, *SMAD9* had previously been associated with disease precipitation and progression due to the onset of manifest PAH in a *Smad9* knock-out mouse model. The *SMAD9* defects in the human studies are thus especially convincing in the light of the mouse model exhibiting clinical and histopathological features of PAH near identical to that observed in patients [98]. Two amino acid substitutions of the type I receptor *BMPR1B* (p.S160N and p.F392L) were recently identified upon the screening of a panel of 43 IPAH patients. *In vitro* and reporter assays indicated that the downstream effect of these variants was to induce increased production of Smad9 associated with or responsible for an increase in transcriptional activity suggestive of a gain-of-function mechanism. As both the human studies, on the basis of functional analyses, when combined with heterozygous loss of Smad9 in the mouse, suggested haploinsufficiency as the molecular mechanism for disease, the conclusions drawn by Chida et al. appear to contradict the preceding three reports. Further functional testing is required to fully elucidate the pathogenicity of these variants [99].

#### 3.4.3. *Caveolin*-1

Homozygous *Caveolin-1* (*Cav1*) knock-out mice develop moderate PAH indicating a role for this gene in the aetiology of disease [100]. Next-generation sequencing technologies, in particular full exome sequencing, have transformed the field of disease gene identification by facilitating the rapid analysis of the entire exome in patient samples. By taking advantage of this methodology Austin et al. successfully identified a second causal gene in familial PAH, namely the human orthologue of *Cav1*. The analysis of 4 members of a multi-generational family led to the detection of a truncating mutation in *CAV1* (c.474delA), a finding which was consolidated by the subsequent demonstration of mutation segregation amongst the other affected members of the family. The *CAV1* variant was observed in unaffected members of this family and this was interpreted as, potentially, being due to incomplete penetrance in a similar vein to mutations of *BMPR2*. Analysis of a replication cohort by Sanger sequencing led to the identification of a second premature truncation (c.473delC) in a single IPAH case lending plausibility to the likelihood of the gene representing a rare inherited risk factor in familial PAH [101].

### 3.5. Modifying Factors Conferring Risk of Disease

Although PAH is an oligogenic disease, family studies demonstrate clinical features reminiscent of a complex disorder, for example, gender bias and reduced penetrance. This concept is lent strength by the existence of several pairs of monozygotic twins discordant for disease [73]. These observations suggest the existence of genetic modifier genes either promoting or protecting against disease. This hypothesis has been subject to much investigation in the form of examination of annotated variation in candidate genes believed to be involved in disease pathogenesis. Amongst others, these include the genes encoding the angiotensin converting enzyme (ACE), the serotonin transporter (at least four independent investigations), endoglin, prostaglandin synthase, and the voltage gated potassium channel subunit Kv1.5 [102–109]. The confounding factor affecting all these studies is the small sample size employed in each case which would not have the statistical power to detect modifying variation of small or moderate effect size. Moreover, validation of initial observations by means of a replication study in an independent cohort has not been attempted. Confirmation by replication studies is now acknowledged as being an essential component of any case control association analysis. In its absence these studies remain incomplete and conclusions drawn, whilst tantalising, have to be treated with some caution. In recent times, the development and wide-spread utilisation of the HapMap resource has revolutionised genome-wide association studies (GWAS). This form of analysis obviates the inefficient interrogation of limited variable loci in a few genes and, combined with the advances in genotyping technology, allows for a rapid examination of several hundred thousand independent loci in large sample sets. The relative lack of success of the candidate gene approach makes a GWAS a much more appealing study design option and one with potential to yield important new insights. Sample size remains an important consideration and due to the low incidence and prevalence of PAH, it is clear global cooperation is required to obtain case groups in excess of 1000 samples.

### 3.6. Animal Models of PH

The Fawn-Hooded rat (FHR) is a spontaneous model of pulmonary hypertension when exposed to mildly hypoxic conditions. While the trait appears to be hereditary there are phenotypic distinctions between PH in this animal and the human condition [110].

Five artificially generated mouse models harbouring *BMPR2* mutation have been reported and routinely employed in the study of the consequences of *BMPR2* disruption on the molecular and phenotypic level. The value of these animal models is twofold. Even in the absence of manifest disease symptoms murine models carrying domain specific *BMPR2* defects provide an invaluable and sustainable source of mutant cell lines that may be employed for interrogation of the BMP pathway in the disease cell and, further, can be characterized on the phenotypic level to gain an insight into the impact of mutation on cellular function. The ultimate goal is that these models go on to recapitulate the disease phenotype, in full or in part, to support the development of research targeted at providing therapeutic options for patients. Two mouse models were generated by utilisation of homologous recombination knock-out methodologies aimed at the removal of exon 2 and exons 4 and 5, respectively. In both instances the mutation proved to be homozygous lethal with the mutant animals dying *in utero*. Whilst both animal models displayed numerous developmental anomalies, cardiac defects particularly of the outflow tract were especially notable in the exon 2 knock-out mouse. In the heterozygous state, these mice were healthy and did not develop measureable features of disease. However, by comparison to wild-type mice, the mutant carriers displayed a significantly higher susceptibility to disease when exposed to environmental risk factors, for example exposure to hypoxia and the inflammatory agent 5-lipooxygenase [111, 112]. A widely used model is a mouse harbouring a point mutation (p.R899X) in exon 12 of the *BMPR2* gene generated by a knock-in strategy. Analogous to the knock-out mice, mutant animals are nonviable in the homozygous condition and asymptomatic in the heterozygous state [113].

Transgenic mouse models are produced through overexpression of a mutant allele introduced as a cDNA construct. As such, they less accurately reflect the genetic architecture of the human mutant carrier. Two transgenic mouse models have been developed to overexpress the point mutations c.504insT and c.2695C>T, driven by the smooth muscle specific promoter SM22, both of which predict incorporation of a premature termination codon [114, 115]. In nature, these mutations would be expected to be lost via the NMD pathway. However, as the NMD process is contingent on the presence of intronic regions, these mutant alleles are spared and expressed in protein form. The presence in the cell of multiple copies of these mutant alleles led to the spontaneous development of aspects of disease. Mice displayed increased pulmonary artery pressure and vessel muscularization. It may be hypothesised that these disease alleles behave in a dominant-negative fashion by acting as a trap for wild type receptors thereby diminishing the available pool of receptors capable of forming active complexes. The precipitation of overt pulmonary hypertension in the transgenic mice lends support for a threshold model of disease. While *BMPR2* haploinsufficiency is a major factor in predisposition to disease, it is not in itself enough to trigger disease in the majority of mutation carriers. Additional factors would appear to be required for disease to manifest and, foremost among these, is the inexplicable loss of wild type receptor in the cells of patients with severe disease. Together with the observations made on the transgenic mouse models, it is likely that a progressive reduction of BMPR-II levels is what is required for a disease state to occur [116].

## 4. Treatment Options in PAH

Treatment for PAH, while much improved in recent times, remains ameliorative and, in general, PAH continues to be a disease distinguished by high morbidity and mortality. All patients are required to undergo a test for vasoreactivity, typically with adenosine or epoprostenol delivered intravenously and/or inhaled nitric oxide. The patient's response is considered positive when the mean pulmonary artery pressure is lowered by 10 mm Hg and remains less than 40 mm Hg with no alteration in cardiac output and systemic pressure. Patients exhibiting a positive response are usually first administered calcium channel blocker treatments; unfortunately the vast majority of patients are nonresponsive and require alternative medical interventions. Calcium channel blockers including diltiazem and nifedipine are delivered at low initial doses and gradually augmented to the maximum level tolerated. After time, the efficacy of this form of treatment decreases and other options need to be explored [117].

### 4.1. Prostacyclin Analogues

The study of cellular pathways disrupted in the progression of PAH has pinpointed three cellular systems that have proven to be amenable to therapeutic intervention. The prostacyclin metabolic pathway is known to be dysregulated in PAH as indicated by reduced expression of prostacyclin synthase in the pulmonary arteries and of prostacyclin metabolites in the urine. Prostacyclin is a multifunctional prostanoid that functions as a powerful vasodilatory agent, an inhibitor of proliferation and platelet aggregation, all hallmark features of the PAH pulmonary circulation [118]. There are three prostacyclin analogues commonly used in patient care, namely epoprostenol, treprostinil, and iloprost. Epoprostenol is continuously administered intravenously at a starting dose of 2 ng/kg/min with an optimal level between 25–40 ng/kg/min [23]. Several substantial studies testify to the efficacy of the treatment in alleviating haemodynamic abnormalities in patients when compared against older, conventional therapies. Improvements were noted in mean arterial pressure, exercise capacity as measured by a 6 minute walk test, and pulmonary vascular resistance. Sitbon et al. conducted a longitudinal study of 178 patients with severe PAH over a five year period and noted survival rates of 85%, 70%, 63%, and 55% over 1, 2, 3, and 5 years [119]. Epoprostenol has been associated with side effects of varying severity that may range from diarrhoea to thrombosis [120]. Treprostinil is also associated with significant improvements in hemodynamic and symptomatic outcomes in patients when infused subcutaneously. As a monotherapy, treprostinil delivers better survival rates than epoprostenol with survival at 88% at one year and 70% at four years by comparison to 69% and 38% [121]. While the adverse effects of this treatment are broadly similar to intravenous injection of epoprostenol, when treprostinil is used intravenously it has potential to lead to a heightened risk of gram negative blood stream infections as well as life threatening catheter infections [122]. Unlike epoprostenol and treprostinil, iloprost is a stable prostacyclin analogue that remains effective for a longer period of time. It also provides the considerable advantage of obviating catheter related complications as it can be inhaled in aerosolized form. A large multi-centre trial, comprising 207 patients with severe PAH (Class III and IV), demonstrated that iloprost therapy resulted in an improvement of at least one functional class [123]. Deleterious effects of iloprost usage are generally mild by comparison to the other commonly employed prostacyclin analogues.

### 4.2. Endothelin-1 Blocking Agents

Endothelin-1 has long been considered to be involved in the aetiology of PAH. It is significantly upregulated in the circulation of PAH patients leading to the development of agents capable of blocking its effects, most prominently bosentan, sitaxsentan, and ambrisentan. Unusually, bosentan is an endothelin antagonist that is capable of blocking both ETA and ETB receptors. Kaplan-Meir survival curves indicated that survival rates of patients treated with bosentan were 96% and 89% in years 1 and 2, respectively [124]. These estimates compared favourably with patients undergoing epoprostenol. Bosentan improves symptoms, haemodynamics, and exercise capacity but can lead to dangerous side effects which include liver problems and hypotension. Male subjects may be at risk of infertility and testicular atrophy [125]. Ambrisentan is a specific ETA receptor antagonist that has been shown by place controlled trials of randomized patients to effect sustained improvements in pulmonary haemodynamics and exercise capacity. While possible testicular atrophy remains a significant side effect, unlike bosentan, the risk of liver damage is low [126].

### 4.3. Combined Therapy

When monotherapy begins to fail to improve symptoms and quality of life in patients a combinatorial strategy is next employed. These, typically, consist of the dual administration of a prostacyclin analogue with an endothelial antagonist. Whilst anecdotal evidence does point to improvements in functional readouts, reduced toxicity, and efficacy of treatment, thus far only a few clinical trials have been run to validate these observations. A placebo controlled study of 405 PAH patients revealed that a combination of bosentan with tadalafil, more known for its usage in treating erectile dysfunction, drove a quantifiable improvement in exercise capacity allied to retardation of clinical worsening [127]. In an analysis of 267 PAH patients, compared to patients treated with placebo while on intravenous epoprostenol, those given sildenafil showed significant improvements in cardiac outputs and mean pulmonary arterial pressure [128].

### 4.4. Therapy for End-Stage Disease

The majority of patients even when benefiting from the highest levels of contemporary PAH therapy will progressively worsen to a critical life-threatening stage. At this time invasive procedures, often atrial septostomy followed by lung transplantation are typically the last remaining options. An atrial septostomy is a procedure designed to introduce a shunt, or conduit, between the right and left atria. This adaptation reduces the blood volume in the right side of the heart and, as a consequence, reduces the work-load in pumping blood to the lungs and improves the ventricular function of the heart. While this procedure provides a measure of clinical stability, it is considered only a precursor to eventual lung transplantation. Lung transplantation, which may be single lung transplantation, double lung transplantation, or heart and lung transplantation, is considered the last therapeutic resort for PAH patients. Following transplantation, patient survival rates have been reported as 66%, 57%, 47%, and 27% at years 1, 3, 5, and 10, respectively [129]. The type of lung transplantation chosen for recipient patients varies internationally from centre to centre.

### 4.5. Current Approaches to Therapy

Recent data released by the Registry to Evaluate Early and Long-term Pulmonary Arterial Hypertension, a longitudinal study of patients from North America, and the French Network on Pulmonary Hypertension, underlines the fact that even with recent improvements in therapeutic protocols, survival figures over three years is still unacceptably modest [130, 131]. Research into PAH has noticeably attempted to address this continuing problem by seeking to translate pathway based studies to therapeutic options. While palliative care has been undoubtedly important in transforming the patient's quality of life, there is increasingly a sense of urgency to employ the growing understanding of defects in the BMP pathway to create directed therapy protocols with the potential to permanently halt or reverse the progression of this disease. To do so, such treatments would be expected to demonstrably remove clinical features of PAH, for example vascular lesions by reverse vascular modelling, and promote the restoration of homeostatic balance between vasoconstrictive and vasodilatory agents.

### 4.6. Gene Therapy

The confounding factors present in the application of gene therapy to human disease are numerous and have stalled the application of this technology to PAH until recently. Complications inherent in gene-based therapies include insertional mutagenesis as well as practical limitations, for example, vector design and delivery, the short duration a beneficial outcome will last and, perhaps, most pertinently in PAH the correct cellular target. Reynolds et al. sought to overcome some of these impediments in a recent study on gene delivery in two experimental rat models of PAH: PAH induced by chronic hypoxia and monocrotaline (MCT)-induced PH. Both rat populations recapitulated the hallmark features of disease, namely raised pulmonary vascular pressure and evidence of vascular modelling. Although neither model harboured *BMPR2* mutation, both had diminished levels of the encoded protein, an as yet unexplained phenomena also observed in PAH patients negative for mutations of the gene. The authors specifically targeted the pulmonary endothelium with a *BMPR2* adenoviral gene delivery vehicle by the linkage of a bispecific antibody to the angiotensin-converting enzyme. BMPR-II expression was detected in and confined to the pulmonary arterioles and alveolar capillaries. Receptor expression was absent in the cardiac sections tested indicating successful and specific delivery to the lung. Introducing the vector to the endothelial layer of pulmonary arteries resulted in a marked improvement in pulmonary haemodynamics including mean pulmonary arterial pressure and right ventricular systolic pressure. Of interest, these changes while positive, were less impressive in the monocrotaline model and did not reach statistical significance. In both models there was clear evidence of a reduction in abnormal vascular remodelling most likely due to a concurrent decrease in the uncontrolled expansion of the endothelial and smooth muscle layers. Recent studies have drawn a clear link between the elevation of TGF-*β* expression and activity in the lung tissue of PAH patients with disease pathogenesis. In the case of the experimental rat models, this disease trait was identified in the MCT treated mouse but was absent in the hypoxia induced model, in itself evidence of the fact that whilst of utility animal models do not fully mirror the human disease state. Nonetheless, the depression of TGF-*β* activity is a much sought after therapeutic goal and the upregulation of BMPR-II signalling activity via adenoviral delivery was found to quench the exuberant TGF-*β* response in the MCT rat model. In follow-up studies conducted on human pulmonary microvascular endothelial cells *in vitro*, this study noted that the restoration of BMPR-II levels and, by extension, rescue of BMP signalling blocked TGF-*β* induced endothelial-mesenchymal transition. This is certainly an important observation but its mechanistic significance in the aetiology of PAH remains to be fully investigated. The primary impact of this particular study is that both rat models, with markedly different initiating factors precipitating disease and substantially distinct phenotypic responses to environmental insults, nonetheless responded to BMPR-II levels being heightened in the correct cell type. These data suggest that the common denominator between different categories of PAH is the level of BMPR-II, likely in the endothelial layer, dropping below a threshold level whereupon the disease state overtly manifests. On a therapy level, this gene delivery option if applicable to humans may benefit a wide spectrum of PH sufferers including those without *BMPR2* mutation [132].

As described previously, prostaglandin enhancement is a vital form of treatment in PAH on account of the disruption of this pathway in the progression of the disease. Yet, this form of treatment loses efficacy over time and, moreover, is associated with several potential complications. Patient quality of life is much impaired when prostacyclin is delivered by use of integrated catheters administering continuous intravenous medication and, additionally, the risk of catheter related infection is significant. PAH was induced in the mouse by prolonged exposure to hypoxic conditions and these animals were employed in a study of the potential feasibility of Prostaglandin I_2_ Synthase (hPGIS) gene delivery in the attenuation and/or reversal of disease symptoms. Moreover, Kataoka et al. investigated the efficiency and effect of an adeno-associated virus (AAV) vector in gene delivery and, further, extended these investigations to different AAV serotypes. Two AAV vectors, type I and type II carrying the hPGIS transcript, were injected into the thigh skeletal tissue of hypoxia induced (10% O_2_) PAH mice which had been exposed to the risk condition for 8 weeks. Both treatments resulted in a decrease of right ventricular systolic pressure, the ratio of right ventricular weight to body weight, and the ratio of RV weight to left ventricular plus septal weight, measures that all reached statistical significance. Additionally, the treatment resulted in a reversal of thickening of the medial wall of pulmonary arterioles. In conclusion, both vector systems were equally effective in halting and, indeed, reversing aspects of the pathogenic PAH phenotype [133].

Yet, it is equally apparent that major obstacles need be overcome. It is unclear, given the present technological constraints, whether this form of treatment is indeed the Holy Grail for sufferers of PAH. The questions that need to be explicated consist of, but are not confined to, whether a gene delivery system that can maintain extended periods of expression is an achievable aim in the foreseeable future. In addition, can a swift change in the cellular environment by, for example, restoring BMPR-II levels, effect a long-term alteration in the framework of the cells comprising the pulmonary circulation? Most importantly, the basic science affected by treatment such as this needs to be fully explored. Are other non-disease systems impacted by treatment and in what way? These and many more considerations remain to be interrogated.

## 5. Future Directions

There is still genetic variation to be uncovered in all forms of PAH. This will be greatly aided by the advent of next-generation sequencing and genotyping systems which will facilitate the discovery of rare and common variation underlying disease. Significant effort needs to be applied in fully elucidating the epidemiology of this condition, in particular to aid genetic counselling of patients. Treatment options, in light of the molecular breakthroughs achieved thus far, must aim higher and execute treatments that become more than simply palliative but, instead, curative.
